# Bis(μ-biphenyl-2,2′-dicarboxyl­ato)bis­[(2,2′-bipyridine)copper(II)]

**DOI:** 10.1107/S160053680802583X

**Published:** 2008-08-16

**Authors:** Hong-Xu Guo, Min Liang, Bin Lin, Qing-Hua Wang, Xi-Zhong Li

**Affiliations:** a Department of Chemistry and Environmental Science, Zhangzhou Normal University, Zhangzhou, Fujian 363000, People’s Republic of China

## Abstract

The title compound, [Cu_2_(C_14_H_8_O_4_)_2_(C_10_H_8_N_2_)_2_], is a centrosymmetric binuclear copper(II) complex, with a Cu⋯Cu separation of 6.136 (16) Å. The Cu atom displays a *cis*-CuN_2_O_2_ square-planar geometry, although two long (> 2.43 Å) Cu⋯O contacts complete a distorted *cis*-CuN_2_O_4_ octa­hedron. Extensive C—H⋯O hydrogen bonds link the mol­ecules into a three-dimensional network.

## Related literature

For related literature, see: Bu *et al.* (2004 ▶); He *et al.* (2007 ▶); Huang *et al.* (2004 ▶); Long *et al.* (2001 ▶); Ma *et al.* (2003 ▶); Rao *et al.* (2004 ▶); Yaghi *et al.* (2003 ▶); Yang *et al.* (2002 ▶); Zhang *et al.* (2004 ▶); Zhu *et al.* (2001 ▶); He & Zhu (2003 ▶).
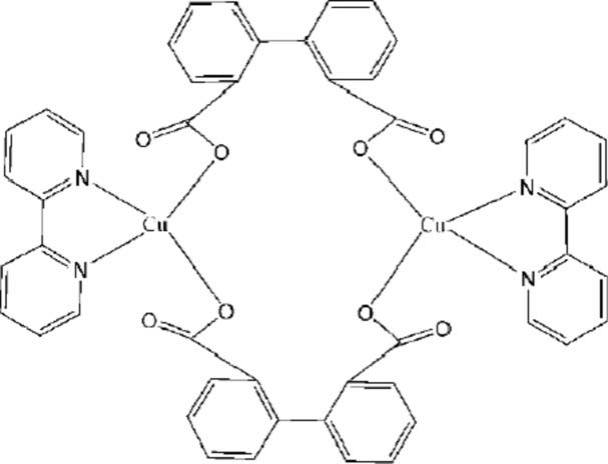


## Experimental

### 

#### Crystal data

[Cu_2_(C_14_H_8_O_4_)_2_(C_10_H_8_N_2_)_2_]
*M* *_r_* = 1839.75
Monoclinic, 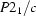

*a* = 11.234 (2) Å
*b* = 13.336 (3) Å
*c* = 15.431 (6) Å
β = 122.16 (2)°
*V* = 1957.1 (9) Å^3^
*Z* = 2
Mo *K*α radiation
μ = 1.15 mm^−1^
*T* = 293 (2) K
0.40 × 0.26 × 0.23 mm

#### Data collection

Siemens SMART CCD area-detector diffractometer
Absorption correction: multi-scan (*SADABS*; Sheldrick, 1996 ▶) *T*_min_ = 0.708, *T*_max_ = 0.771
18687 measured reflections
4472 independent reflections
3708 reflections with *I* > 2σ(*I*)
*R*_int_ = 0.046

#### Refinement

*R*[*F* ^2^ > 2σ(*F* ^2^)] = 0.036
*wR*(*F* ^2^) = 0.118
*S* = 1.03
4472 reflections
280 parameters
H-atom parameters constrained
Δρ_max_ = 0.29 e Å^−3^
Δρ_min_ = −0.60 e Å^−3^

### 

Data collection: *SMART* (Siemens, 1994 ▶); cell refinement: *SAINT* (Siemens, 1994 ▶); data reduction: *SAINT*; program(s) used to solve structure: *SHELXTL* (Sheldrick, 2008 ▶); program(s) used to refine structure: *SHELXTL*; molecular graphics: *SHELXTL*; software used to prepare material for publication: *SHELXTL*.

## Supplementary Material

Crystal structure: contains datablocks I, global. DOI: 10.1107/S160053680802583X/om2255sup1.cif

Structure factors: contains datablocks I. DOI: 10.1107/S160053680802583X/om2255Isup2.hkl

Additional supplementary materials:  crystallographic information; 3D view; checkCIF report

## Figures and Tables

**Table:** 

Cu1—O1	1.9640 (15)
Cu1—O4^i^	1.9725 (16)
Cu1—N2	1.9814 (19)
Cu1—N1	1.9897 (19)
Cu1—O2	2.434 (2)
Cu1—O3^i^	2.557 (2)

**Table:** 

O1—Cu1—O4^i^	93.92 (7)
O1—Cu1—N2	162.77 (7)
O4^i^—Cu1—N2	95.38 (8)
O1—Cu1—N1	94.56 (7)
O4^i^—Cu1—N1	160.15 (7)
N2—Cu1—N1	81.35 (8)

Symmetry code: (i) 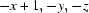.

**Table 2:** Hydrogen-bond geometry (Å, °)

*D*—H⋯*A*	*D*—H	H⋯*A*	*D*⋯*A*	*D*—H⋯*A*
C1—H1*A*⋯O1	0.93	2.58	3.081 (3)	114
C4—H4*A*⋯O4^ii^	0.93	2.59	3.378 (3)	143
C5—H5*A*⋯O4^ii^	0.93	2.51	3.304 (4)	144
C6—H6*A*⋯O3^iii^	0.93	2.25	3.162 (3)	166
C16—H16*A*⋯O2^iv^	0.93	2.48	3.192 (3)	133
C19—H19*A*⋯O4	0.93	2.45	2.761 (3)	100

Symmetry codes: (ii) 
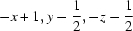
; (iii) 
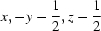
; (iv) 
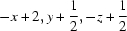
.

**Fig. 1.**
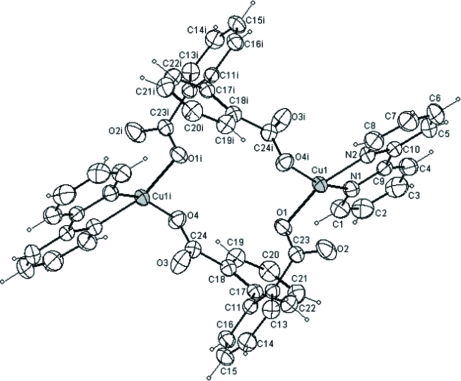
View of the structure of compound (I), showing the atom-labelling scheme. Displacement ellipsoids are drawn at the 35% probability level; H-atoms are shown as small spheres of arbitrary radius.

**Fig. 2.**
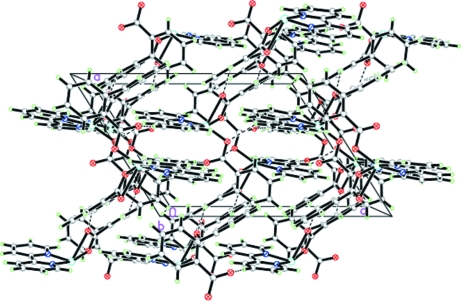
View of the 3D hydrogen-bonded network in the packing of the title compound.The packing is viewed along the b axis; C—H···O interactions are shown as dashed lines.

## supplementary crystallographic information

### Comment

Design and assembly of metal-involved supramolecular architectures are currently of great interest in the field of supramolecular chemistry and crystal engineering because they can provide novel topology and functional materials (Yaghi *et al.*,2003; Rao *et al.*,2004). During the past decades, extensive efforts have been focused on the design and assembly of such kinds of supramolecular architectures (Huang *et al.*,2004; Zhang *et al.*, 2004). By precisely selecting the modular building unit, chemists now have successfully synthesized a great variety of one-dimensional, two-dimensional, and three-dimensional supramolecular architectures (Bu *et al.*, 2004; Ma *et al.*, 2003; Yang *et al.*, 2002; Long *et al.*, 2001). Binuclear copper(II) complexes have been intensely investigated owing to their potential application as magnetic materials and catalysts (Zhu *et al.*, 2001).In this work, we employed H_2_dpa (dpa = diphenyl-2,2'-dicarboxylato dianion) and 2,2'-bipyridine(bipy) ligands for producing a binuclear complex, [Cu_2_(C_14_H_8_O_4_)_2_(C_10_H_8_N_2_)_2_].

The compound contains a centrosymmetric binuclear complex. The copper(II) atom in the title compound adopts a distorted square geometry (Table 1, Fig. 1). The bipy ligand shows its classical bidentate coordination mode, with a similar Cu—N bond length to that the related complex [Cu_2_(C_14_H_8_O_4_)_2_(C_10_H_8_N_2_)_2_].4H_2_O (He *et al.*, 2007). The dpa ligand adopts a µ-bridged coordination and the dihedral angle between its aromatic rings is 78.27°. As well as the short Cu—O bonds, two long Cu—O (Cu(1)—O(2): 2.434 (44) Å; Cu(1)—O(3):2.557 (31) Å) contacts that might be regarded as secondary bonds (He & Zhu, 2003) complete a distorted octahedron. The Cu···Cu^i^ (i = 1 - *x*, -*y*, -*z*) distance bridged by the dpa ligands is 6.136 (16) Å. Extensive C—H···O hydrogen bonds link molecules into a three-dimensional network.(Table 2, Fig.2).

### Experimental

A solution of Cu(NO_3_)_2_.6H_2_O(0.0705 g) in 5 ml of water was added dropwise under continuous stirring to an aqueous solution (5 ml) of diphenyl-2,2'-dicarboxylic acid (0.0734 g) and 2,2'-bipyridine (0.0312 g). The resulting mixture was then transferred into a 25 ml Teflon-lined stainless steel vessel, which was sealed and heated to 423 K for 72 h, then cooled to room temperature. The block blue single crystals were obtained.

### Refinement

The phenyl H atoms were positioned geometrically and allowed to ride during subsequent refinement, with C—H = 0.93 Å and *U*_iso_(H) = 1.2*U*_eq_(C).

### Crystal data

**Table:**

[Cu_2_(C_14_H_8_O_4_)_2_(C_10_H_8_N_2_)_2_]	*F*_000_ = 940
*M**_r_* = 1839.75	*D*_x_ = 1.561 Mg m^−^^3^
Monoclinic, *P*2_1_/*c*	Mo *K*α radiation λ = 0.71073 Å
Hall symbol: -P 2ybc	Cell parameters from 19150 reflections
*a* = 11.234 (2) Å	θ = 3.1–27.4º
*b* = 13.336 (3) Å	µ = 1.15 mm^−^^1^
*c* = 15.431 (6) Å	*T* = 293 (2) K
β = 122.16 (2)º	Block, blue
*V* = 1957.1 (9) Å^3^	0.40 × 0.26 × 0.23 mm
*Z* = 2	

### Data collection

**Table:**

Siemens SMART CCD area-detector diffractometer	4472 independent reflections
Radiation source: fine-focus sealed tube	3708 reflections with *I* > 2σ(*I*)
Monochromator: graphite	*R*_int_ = 0.046
*T* = 293(2) K	θ_max_ = 27.4º
ω scans	θ_min_ = 3.1º
Absorption correction: multi-scan(SADABS; Sheldrick, 1996)	*h* = −14→14
*T*_min_ = 0.708, *T*_max_ = 0.771	*k* = −17→17
18687 measured reflections	*l* = −18→19

### Refinement

**Table:**

Refinement on *F*^2^	Secondary atom site location: difference Fourier map
Least-squares matrix: full	Hydrogen site location: inferred from neighbouring sites
*R*[*F*^2^ > 2σ(*F*^2^)] = 0.036	H-atom parameters constrained
*wR*(*F*^2^) = 0.118	*w* = 1/[σ^2^(*F*_o_^2^) + (0.08*P*)^2^] where *P* = (*F*_o_^2^ + 2*F*_c_^2^)/3
*S* = 1.04	(Δ/σ)_max_ = 0.001
4472 reflections	Δρ_max_ = 0.29 e Å^−^^3^
280 parameters	Δρ_min_ = −0.60 e Å^−^^3^
Primary atom site location: structure-invariant direct methods	Extinction correction: none

### Special details

**Table:**

Geometry. All e.s.d.'s (except the e.s.d. in the dihedral angle between two l.s. planes) are estimated using the full covariance matrix. The cell e.s.d.'s are taken into account individually in the estimation of e.s.d.'s in distances, angles and torsion angles; correlations between e.s.d.'s in cell parameters are only used when they are defined by crystal symmetry. An approximate (isotropic) treatment of cell e.s.d.'s is used for estimating e.s.d.'s involving l.s. planes.
Refinement. Refinement of *F*^2^ against ALL reflections. The weighted *R*-factor *wR* and goodness of fit *S* are based on *F*^2^, conventional *R*-factors *R* are based on *F*, with *F* set to zero for negative *F*^2^. The threshold expression of *F*^2^ > σ(*F*^2^) is used only for calculating *R*-factors(gt) *etc*. and is not relevant to the choice of reflections for refinement. *R*-factors based on *F*^2^ are statistically about twice as large as those based on *F*, and *R*- factors based on ALL data will be even larger.

### Fractional atomic coordinates and isotropic or equivalent isotropic displacement parameters (Å^2^)

**Table:**

	*x*	*y*	*z*	*U*_iso_*/*U*_eq_	
Cu1	0.63909 (3)	−0.160660 (19)	−0.059678 (18)	0.02914 (12)	
O1	0.70349 (16)	−0.04363 (11)	0.03157 (11)	0.0354 (4)	
O2	0.8638 (2)	−0.16096 (12)	0.10287 (15)	0.0530 (5)	
O3	0.62962 (19)	0.15631 (14)	0.16045 (14)	0.0480 (5)	
O4	0.47467 (16)	0.21452 (12)	0.00754 (11)	0.0374 (4)	
N1	0.68794 (18)	−0.10223 (14)	−0.15551 (13)	0.0320 (4)	
N2	0.63566 (18)	−0.28542 (14)	−0.13069 (14)	0.0331 (4)	
C1	0.7186 (2)	−0.00573 (19)	−0.15928 (18)	0.0406 (5)	
H1A	0.7227	0.0390	−0.1115	0.049*	
C2	0.7441 (3)	0.0289 (2)	−0.2320 (2)	0.0504 (7)	
H2A	0.7664	0.0959	−0.2327	0.060*	
C3	0.7361 (3)	−0.0368 (2)	−0.3032 (2)	0.0531 (7)	
H3A	0.7514	−0.0145	−0.3536	0.064*	
C4	0.7051 (3)	−0.1367 (2)	−0.29961 (19)	0.0462 (6)	
H4A	0.7002	−0.1823	−0.3470	0.055*	
C5	0.6486 (3)	−0.3529 (2)	−0.2682 (2)	0.0487 (7)	
H5A	0.6566	−0.3429	−0.3246	0.058*	
C6	0.6319 (3)	−0.4472 (2)	−0.2420 (3)	0.0585 (8)	
H6A	0.6296	−0.5022	−0.2800	0.070*	
C7	0.6184 (3)	−0.4608 (2)	−0.1591 (2)	0.0540 (7)	
H7A	0.6097	−0.5248	−0.1392	0.065*	
C8	0.6181 (3)	−0.37789 (19)	−0.1068 (2)	0.0444 (6)	
H8A	0.6053	−0.3865	−0.0525	0.053*	
C9	0.6815 (2)	−0.16746 (17)	−0.22479 (17)	0.0332 (5)	
C10	0.6534 (2)	−0.27237 (17)	−0.20994 (17)	0.0337 (5)	
C11	0.8922 (2)	0.08967 (16)	0.20040 (15)	0.0278 (4)	
C12	0.8946 (2)	−0.01519 (16)	0.20175 (15)	0.0281 (4)	
C13	0.9639 (2)	−0.06631 (17)	0.29523 (16)	0.0346 (5)	
H13A	0.9681	−0.1360	0.2959	0.042*	
C14	1.0260 (2)	−0.0142 (2)	0.38647 (16)	0.0405 (5)	
H14A	1.0703	−0.0486	0.4484	0.049*	
C15	1.0221 (2)	0.0895 (2)	0.38529 (17)	0.0419 (6)	
H15A	1.0636	0.1249	0.4466	0.050*	
C16	0.9568 (2)	0.14064 (17)	0.29350 (18)	0.0365 (5)	
H16A	0.9559	0.2104	0.2937	0.044*	
C17	0.8383 (2)	0.14901 (14)	0.10351 (17)	0.0282 (4)	
C18	0.7102 (2)	0.19898 (15)	0.04996 (16)	0.0292 (4)	
C19	0.6773 (2)	0.25507 (18)	−0.03716 (18)	0.0369 (5)	
H19A	0.5920	0.2890	−0.0727	0.044*	
C20	0.7682 (3)	0.26123 (18)	−0.07140 (19)	0.0412 (5)	
H20A	0.7446	0.2991	−0.1290	0.049*	
C21	0.8951 (2)	0.21017 (18)	−0.01881 (19)	0.0400 (5)	
H21A	0.9573	0.2131	−0.0412	0.048*	
C22	0.9287 (3)	0.15476 (17)	0.06719 (19)	0.0366 (5)	
H22A	1.0137	0.1204	0.1018	0.044*	
C23	0.8187 (2)	−0.07766 (15)	0.10614 (16)	0.0300 (4)	
C24	0.5999 (2)	0.18908 (16)	0.07706 (17)	0.0309 (4)	

### Atomic displacement parameters (Å^2^)

**Table:**

	*U*^11^	*U*^22^	*U*^33^	*U*^12^	*U*^13^	*U*^23^
Cu1	0.03431 (18)	0.02951 (18)	0.02666 (17)	−0.00125 (10)	0.01828 (13)	−0.00287 (9)
O1	0.0386 (8)	0.0330 (8)	0.0287 (8)	0.0015 (7)	0.0138 (7)	−0.0053 (6)
O2	0.0586 (12)	0.0359 (10)	0.0433 (10)	0.0155 (8)	0.0128 (9)	−0.0077 (7)
O3	0.0446 (10)	0.0630 (12)	0.0467 (11)	0.0160 (8)	0.0313 (9)	0.0260 (8)
O4	0.0328 (8)	0.0494 (10)	0.0328 (8)	0.0021 (7)	0.0193 (7)	0.0047 (7)
N1	0.0321 (9)	0.0371 (10)	0.0286 (9)	−0.0026 (8)	0.0174 (8)	−0.0026 (8)
N2	0.0323 (9)	0.0339 (10)	0.0325 (9)	0.0008 (8)	0.0168 (8)	−0.0031 (8)
C1	0.0441 (13)	0.0396 (13)	0.0410 (13)	−0.0049 (11)	0.0247 (11)	0.0004 (10)
C2	0.0523 (15)	0.0506 (16)	0.0521 (16)	−0.0115 (13)	0.0304 (13)	0.0044 (12)
C3	0.0485 (15)	0.075 (2)	0.0423 (14)	−0.0104 (14)	0.0288 (12)	0.0046 (13)
C4	0.0412 (13)	0.0684 (17)	0.0339 (12)	−0.0086 (12)	0.0233 (11)	−0.0095 (12)
C5	0.0477 (15)	0.0549 (17)	0.0515 (16)	−0.0021 (12)	0.0317 (13)	−0.0170 (12)
C6	0.0578 (17)	0.0476 (16)	0.074 (2)	−0.0064 (13)	0.0380 (16)	−0.0293 (15)
C7	0.0522 (16)	0.0322 (13)	0.076 (2)	−0.0053 (12)	0.0327 (15)	−0.0113 (12)
C8	0.0475 (14)	0.0354 (13)	0.0500 (15)	−0.0028 (11)	0.0259 (12)	−0.0028 (11)
C9	0.0253 (10)	0.0467 (13)	0.0273 (11)	−0.0003 (9)	0.0138 (9)	−0.0040 (9)
C10	0.0268 (10)	0.0414 (13)	0.0313 (11)	−0.0001 (9)	0.0144 (9)	−0.0076 (9)
C11	0.0261 (9)	0.0282 (10)	0.0298 (10)	0.0002 (8)	0.0154 (8)	−0.0003 (8)
C12	0.0282 (10)	0.0304 (11)	0.0269 (10)	0.0008 (8)	0.0154 (8)	0.0001 (8)
C13	0.0383 (12)	0.0330 (11)	0.0337 (11)	0.0039 (9)	0.0199 (10)	0.0056 (9)
C14	0.0416 (13)	0.0515 (14)	0.0261 (11)	0.0053 (11)	0.0166 (10)	0.0062 (10)
C15	0.0418 (12)	0.0521 (15)	0.0258 (11)	0.0020 (11)	0.0139 (10)	−0.0096 (10)
C16	0.0386 (12)	0.0322 (11)	0.0365 (12)	0.0003 (9)	0.0184 (10)	−0.0055 (9)
C17	0.0328 (11)	0.0243 (10)	0.0302 (11)	−0.0037 (8)	0.0186 (9)	−0.0015 (8)
C18	0.0345 (11)	0.0242 (10)	0.0320 (11)	−0.0024 (9)	0.0198 (9)	0.0004 (8)
C19	0.0403 (12)	0.0332 (12)	0.0382 (12)	0.0040 (10)	0.0215 (10)	0.0099 (10)
C20	0.0530 (14)	0.0362 (12)	0.0422 (13)	−0.0049 (11)	0.0305 (11)	0.0083 (10)
C21	0.0462 (13)	0.0398 (13)	0.0487 (14)	−0.0066 (11)	0.0352 (12)	0.0014 (11)
C22	0.0339 (12)	0.0389 (13)	0.0394 (13)	−0.0010 (9)	0.0211 (10)	0.0007 (9)
C23	0.0357 (11)	0.0280 (11)	0.0294 (10)	−0.0010 (9)	0.0194 (9)	−0.0010 (8)
C24	0.0346 (11)	0.0257 (10)	0.0360 (11)	0.0013 (9)	0.0211 (9)	0.0030 (9)

### Geometric parameters (Å, °)

**Table:**

Cu1—O1	1.9640 (15)	C6—H6A	0.9300
Cu1—O4^i^	1.9725 (16)	C7—C8	1.370 (4)
Cu1—N2	1.9814 (19)	C7—H7A	0.9300
Cu1—N1	1.9897 (19)	C8—H8A	0.9300
Cu1—O2	2.434 (2)	C9—C10	1.479 (3)
Cu1—C23	2.519 (2)	C11—C16	1.394 (3)
Cu1—O3^i^	2.557 (2)	C11—C12	1.399 (3)
Cu1—C24^i^	2.580 (2)	C11—C17	1.505 (3)
O1—C23	1.273 (2)	C12—C13	1.399 (3)
O2—C23	1.233 (3)	C12—C23	1.503 (3)
O3—C24	1.225 (3)	C13—C14	1.381 (3)
O3—Cu1^i^	2.5567 (19)	C13—H13A	0.9300
O4—C24	1.280 (3)	C14—C15	1.383 (4)
O4—Cu1^i^	1.9725 (16)	C14—H14A	0.9300
N1—C1	1.342 (3)	C15—C16	1.380 (3)
N1—C9	1.350 (3)	C15—H15A	0.9300
N2—C8	1.331 (3)	C16—H16A	0.9300
N2—C10	1.351 (3)	C17—C18	1.390 (3)
C1—C2	1.377 (3)	C17—C22	1.399 (3)
C1—H1A	0.9300	C18—C19	1.405 (3)
C2—C3	1.372 (4)	C18—C24	1.509 (3)
C2—H2A	0.9300	C19—C20	1.379 (3)
C3—C4	1.385 (4)	C19—H19A	0.9300
C3—H3A	0.9300	C20—C21	1.387 (3)
C4—C9	1.377 (3)	C20—H20A	0.9300
C4—H4A	0.9300	C21—C22	1.384 (3)
C5—C6	1.365 (4)	C21—H21A	0.9300
C5—C10	1.383 (3)	C22—H22A	0.9300
C5—H5A	0.9300	C24—Cu1^i^	2.580 (2)
C6—C7	1.378 (5)		
			
O1—Cu1—O4^i^	93.92 (7)	C6—C7—H7A	120.8
O1—Cu1—N2	162.77 (7)	N2—C8—C7	122.5 (3)
O4^i^—Cu1—N2	95.38 (8)	N2—C8—H8A	118.8
O1—Cu1—N1	94.56 (7)	C7—C8—H8A	118.8
O4^i^—Cu1—N1	160.15 (7)	N1—C9—C4	121.3 (2)
N2—Cu1—N1	81.35 (8)	N1—C9—C10	114.4 (2)
O1—Cu1—O2	58.55 (6)	C4—C9—C10	124.3 (2)
O4^i^—Cu1—O2	96.94 (8)	N2—C10—C5	121.0 (2)
N2—Cu1—O2	105.83 (7)	N2—C10—C9	114.12 (19)
N1—Cu1—O2	102.80 (8)	C5—C10—C9	124.9 (2)
O1—Cu1—C23	29.83 (6)	C16—C11—C12	118.49 (19)
O4^i^—Cu1—C23	95.07 (7)	C16—C11—C17	118.75 (19)
N2—Cu1—C23	134.42 (7)	C12—C11—C17	122.34 (18)
N1—Cu1—C23	101.19 (7)	C11—C12—C13	119.90 (19)
O2—Cu1—C23	28.76 (6)	C11—C12—C23	122.91 (18)
O1—Cu1—O3^i^	106.45 (7)	C13—C12—C23	117.11 (19)
O4^i^—Cu1—O3^i^	56.28 (6)	C14—C13—C12	120.5 (2)
N2—Cu1—O3^i^	90.78 (7)	C14—C13—H13A	119.7
N1—Cu1—O3^i^	104.04 (7)	C12—C13—H13A	119.7
O2—Cu1—O3^i^	150.22 (7)	C13—C14—C15	119.7 (2)
C23—Cu1—O3^i^	130.98 (7)	C13—C14—H14A	120.2
O1—Cu1—C24^i^	99.04 (7)	C15—C14—H14A	120.2
O4^i^—Cu1—C24^i^	28.92 (6)	C16—C15—C14	120.2 (2)
N2—Cu1—C24^i^	96.11 (7)	C16—C15—H15A	119.9
N1—Cu1—C24^i^	131.58 (7)	C14—C15—H15A	119.9
O2—Cu1—C24^i^	123.91 (8)	C15—C16—C11	121.2 (2)
C23—Cu1—C24^i^	113.37 (7)	C15—C16—H16A	119.4
O3^i^—Cu1—C24^i^	27.59 (6)	C11—C16—H16A	119.4
C23—O1—Cu1	100.02 (13)	C18—C17—C22	118.57 (19)
C23—O2—Cu1	79.48 (13)	C18—C17—C11	125.76 (19)
C24—O3—Cu1^i^	77.28 (13)	C22—C17—C11	115.66 (19)
C24—O4—Cu1^i^	102.92 (13)	C17—C18—C19	119.05 (19)
C1—N1—C9	119.4 (2)	C17—C18—C24	122.42 (19)
C1—N1—Cu1	125.93 (16)	C19—C18—C24	118.37 (19)
C9—N1—Cu1	114.61 (15)	C20—C19—C18	121.8 (2)
C8—N2—C10	119.0 (2)	C20—C19—H19A	119.1
C8—N2—Cu1	125.93 (17)	C18—C19—H19A	119.1
C10—N2—Cu1	115.06 (15)	C19—C20—C21	119.1 (2)
N1—C1—C2	121.8 (2)	C19—C20—H20A	120.4
N1—C1—H1A	119.1	C21—C20—H20A	120.4
C2—C1—H1A	119.1	C22—C21—C20	119.6 (2)
C3—C2—C1	119.0 (3)	C22—C21—H21A	120.2
C3—C2—H2A	120.5	C20—C21—H21A	120.2
C1—C2—H2A	120.5	C21—C22—C17	121.9 (2)
C2—C3—C4	119.5 (2)	C21—C22—H22A	119.1
C2—C3—H3A	120.2	C17—C22—H22A	119.1
C4—C3—H3A	120.2	O2—C23—O1	121.8 (2)
C9—C4—C3	119.0 (2)	O2—C23—C12	120.6 (2)
C9—C4—H4A	120.5	O1—C23—C12	117.53 (18)
C3—C4—H4A	120.5	O2—C23—Cu1	71.76 (13)
C6—C5—C10	119.1 (3)	O1—C23—Cu1	50.14 (10)
C6—C5—H5A	120.4	C12—C23—Cu1	165.85 (15)
C10—C5—H5A	120.4	O3—C24—O4	122.5 (2)
C5—C6—C7	119.8 (2)	O3—C24—C18	121.2 (2)
C5—C6—H6A	120.1	O4—C24—C18	116.28 (18)
C7—C6—H6A	120.1	O3—C24—Cu1^i^	75.13 (13)
C8—C7—C6	118.5 (3)	O4—C24—Cu1^i^	48.16 (10)
C8—C7—H7A	120.8	C18—C24—Cu1^i^	161.10 (15)

Symmetry codes: (i) −*x*+1, −*y*, −*z*.

### Hydrogen-bond geometry (Å, °)

**Table:**

*D*—H···*A*	*D*—H	H···*A*	*D*···*A*	*D*—H···*A*
C1—H1A···O1	0.93	2.58	3.081 (3)	114
C4—H4A···O4^ii^	0.93	2.59	3.378 (3)	143
C5—H5A···O4^ii^	0.93	2.51	3.304 (4)	144
C6—H6A···O3^iii^	0.93	2.25	3.162 (3)	166
C16—H16A···O2^iv^	0.93	2.48	3.192 (3)	133
C19—H19A···O4	0.93	2.45	2.761 (3)	100

Symmetry codes: (ii) −*x*+1, *y*−1/2, −*z*−1/2; (iii) *x*, −*y*−1/2, *z*−1/2; (iv) −*x*+2, *y*+1/2, −*z*+1/2.
